# Cytotoxicity Testing of Temporary Luting Cements with Two- and Three-Dimensional Cultures of Bovine Dental Pulp-Derived Cells

**DOI:** 10.1155/2013/910459

**Published:** 2013-07-28

**Authors:** Hayriye Esra Ülker, Mustafa Ülker, Hasan Önder Gümüş, Muhammet Yalçın, Abdulkadir Şengün

**Affiliations:** ^1^Department of Restorative Dentistry, Faculty of Dentistry, Selcuk University, 42075 Konya, Turkey; ^2^Department of Prosthodontics, Faculty of Dentistry, Erciyes University, 38039 Kayseri, Turkey; ^3^Department of Restorative Dentistry, Faculty of Dentistry, Inonu University, 44280 Malatya, Turkey; ^4^Department of Restorative Dentistry, Faculty of Dentistry, Kırıkkale University, 71100 Kırıkkale, Turkey

## Abstract

This study evaluated the cytotoxicity of eugenol-containing and eugenol-free temporary luting cements. For cytotoxicity testing, bovine pulp-derived cells transfected with Simian virus 40 Large T antigen were exposed to extracts of eugenol-containing (Rely X Temp E) and eugenol-free (Provicol, PreVISION CEM, and Rely X Temp NE) temporary luting cements for 24 h. The cytotoxicity of the same materials was also evaluated in a dentin barrier test device using three-dimensional cell cultures of bovine pulp-derived cells. The results of the cytotoxicity studies with two-dimensional cultures of bovine dental pulp-derived cells revealed that cell survival with the extracts of Rely X Temp E, Provicol, PreVISION CEM, and Rely X Temp NE was 89.1%, 84.9%, 92.3%, and 66.8%, respectively. Rely X Temp NE and Provicol showed cytotoxic effects on bovine dental pulp-derived cells (*P* < 0.05). The results of the dentin barrier test revealed that cell survival with the above-mentioned temporary cement was 101.5%, 91.9%, 93.5%, and 90.6%, respectively. None of the temporary luting cements significantly reduced cell survival compared with the negative control in the dentin barrier test (*P* > 0.05). Biologically active materials released from temporary luting cements may not influence the dentine-pulp complex if the residual dentine layer is at least 0.5 mm thick.

## 1. Introduction

Indirect restorations generally require less chair side time and provide better proximal contacts, esthetics, tooth morphology, and marginal accuracy than direct restorations [1, 2]. However, as indirect procedures require multiple appointments, the use of temporary restorations and temporary luting cements is necessary to cover the prepared part of the tooth and to protect the pulp from external stimuli, before a patient's cosmetic and functional needs are fully restored [2, 3]. Sometimes temporary restorations may have to function for an extended period of time as a result of unforeseen events such as laboratory delays or patient unavailability [4, 5]. Moreover, the provisional cementation of permanent restorations by temporary luting cements is widely practiced for a variety of clinical reasons, including the desire to make further functional, occlusal, and esthetic adjustments [5, 6].

Therefore, provisional or definitive restorations may be placed with temporary luting cement for an extended time period. During this period, the abutments need the best possible biological and mechanical protection. This ensures that the vitality of the pulp and the integrity of mineralized tissues are preserved. As temporary luting cements come into close and relatively prolonged contact with the vital dentin-pulp complex, their influence on pulp tissue is very important. Thus, the biocompatibility of temporary luting cements is a relevant aspect of these materials' clinical success. The biological safety of permanent conventional and adhesive dental luting cements has been studied extensively, and prior findings demonstrate varying degrees of biological effects [7–12]. However reports on the biological safety of temporary luting cements are still very rare. In a recent *in vivo* study, Bagis et al. demonstrated that temporary luting cements altered the expression of endothelial cell adhesion molecules in the dental pulp [13].

To evaluate the cytotoxicity of temporary luting cements on bovine dental pulp-derived cells completely, this study first included an evaluation of cytotoxicity of several temporary luting cements (RelyX Temp E, Provicol, PreVISION CEM, and RelyX Temp NE) on two-dimensional monolayer cultures of bovine dental pulp-derived cells. In addition, the cytotoxicity of temporary luting cements was also evaluated in a dentin barrier test device using three-dimensional cultures of bovine dental pulp-derived cells to mimic an *in vivo* situation. The null hypothesis of this study was that temporary luting cements are not cytotoxic to both two-dimensional monolayer cultures and the three-dimensional cultures of bovine dental pulp-derived cells. 

## 2. Materials and Methods

### 2.1. Preparation of Extracts and Cytotoxicity Testing Using Bovine Dental Pulp-Derived Cells

Three eugenol-free temporary luting cements were tested: Provicol (Voco GmbH, Cuxhaven, Germany), PreVISION CEM (Heraeus Kulzer GmbH & Co. KG, Hanau, Germany), and RelyX Temp NE (3 M ESPE, Seefeld, Germany). Additionally, one eugenol-containing temporary luting cement was included into this study: RelyX Temp E (3 M ESPE, Seefeld, Germany). The temporary luting cement materials, their composition, their batch numbers, and their manufacturers are described in Table 1.

Samples of the materials were prepared according to manufacturers' directions under aseptic conditions to prevent the risk of biological contamination during the cytotoxicity testing. Temporary cements were prepared in sterile Teflon rings (5 mm in diameter, 2 mm high). After 10 minutes of setting tested materials in a humidified atmosphere of 95% air/5% CO_2_ at 37°C, seven specimens per material were transferred into one insert (Millipore: 0.4 *μ*m pore size, 30 mm diameter) of a six-well plate. The test specimens were covered with a 3 mL cell culture medium [*α*-MEM supplemented with 20% FBS, Geneticin, and penicillin/streptomycin] and incubated for 24 h in a humidified atmosphere of 95% air/5% CO_2_ at 37°C. Thus, extracts of the test specimens were prepared at a ratio of 91.6 mm^2^ sample surface area/mL cell culture medium following the recommendations of ISO [14]. 

Clonal SV40 large T-antigen-transfected bovine dental pulp-derived cells were routinely cultivated in *α*-MEM supplemented with 20% FBS, penicillin (150 IU/mL), Geneticin (0.1 mg/mL), and streptomycin (150 *μ*g/mL) at 37°C and 5% CO_2_, as previously described [15]. Cells within passages 19 to 23 were used.

 The bovine dental pulp-derived cells were seeded at a density of 5 × 10^3^ into each well of a 96-well plate and incubated for 24 hours at 37°C. Then, the cell cultures were exposed to either 200 *μ*L original extracts of the test specimens or a medium as a negative control. Cell viability was than determined by enzyme activity (MTT assay). Cell cultures were washed with phosphate-buffered saline (PBS). Subsequently, 200 *μ*L aliquots of freshly prepared MTT solution (0.5 mg/mL in growth medium) was added to each well and incubated for 2 h at 37°C. The cells were then washed two times with PBS. The blue formazan precipitate was extracted from the mitochondria by using 200 *μ*L of dimethyl sulfoxide on a shaker at room temperature for 30 min. The absorption at 540 nm (OD_540_) was determined spectrophotometrically. Twelve wells were used for each specimen in two independent experiments (*n* = 24). Optical density readings detected in cultures exposed to extracts were normalized to untreated control cultures (=100%). The data were normally distributed. Results were statistically analyzed by one-way ANOVA followed by the Tukey-HSD test for post hoc comparisons (*α* = 0.05; SPSS version 13.0; SPSS, Chicago, IL).

### 2.2. Dentin Barrier Test

The cytotoxicity of the temporary luting cements was also evaluated in a dentin barrier test device using three-dimensional cell cultures of bovine dental pulp-derived cells. Three-dimensional cultures of bovine dental pulp-derived cells were prepared as previously described [9, 16]. 

Polyamide meshes (0.5 cm^2^; Reichelt Chemietechnik, Heidelberg, Germany) were immersed in 0.1 M of acetic acid for 30 min, washed three times with PBS, and air dried. Next, meshes were coated with fibronectin (0.03 mg/mL; Sigma, Deisenhofen, Germany) and air dried. Cell culture inserts (Millipore, Eschborn, Germany) were placed into six-well plates with 1.25 mL of growth medium per well. The meshes were placed on the inserts, and 20 *μ*L of cell suspension (4 × 10^6^ cells/mL) was seeded on them. After 48 h of incubation (37°C, 5% CO_2_, 100% humidity), the meshes were transferred to 24-well plates and incubated until they were used for cytotoxicity experiments (14 ± 2 days). The culture medium (growth medium supplemented with 50 g/mL of ascorbic acid) was changed three times a week. 

A commercially available cell culture perfusion chamber (Minucells & Minutissue GmbH, Bad Abbach, Germany) made of polycarbonate with a base of 40 × 40 mm and a height of 36 mm was modified. The three-dimensional cultures were placed on a dentin disc held in place by a special biocompatible stainless-steel holder, resulting in a dentin barrier test situation. The dentin disc (500 ± 20 *μ*m thick) was cut from a bovine incisor, etched on the pulpal side with 50% citric acid for 30 s, and sterilized by autoclaving as described [16]. Thus, the cell culture chamber was separated into two compartments by the dentin disc. The cell culture tissues were placed in direct contact with the etched side of the dentin disk and held in place by the stainless-steel holder. All chambers were perfused with 0.3 mL of assay medium (growth medium with 5.96 g/L HEPES buffer, Merck, Germany) per hour for 24 h at 37°C. Perfusion was switched off; test materials were introduced into the upper compartment in direct contact with the “cavity” side of the dentin disc. Test materials were applied according to the manufacturers' instructions. A nontoxic polyvinylsiloxane impression material (President, Coltene) was used as a negative control (100% cell viability).

Cytotoxicity of test materials was recorded after the pulpal part of the *in vitro* pulp chamber was perfused with the cell culture medium (2 mL/h) for 24 h of incubation at 37°C. Five chambers were used for each material in two independent experiments (*n* = 10); after 24 h of incubation, the vitality of the cultures was determined using the MTT assay.

Cell viability of the three-dimensional cultures was determined by enzyme activity (MTT assay). The tissues were removed from the pulp chambers, placed into 24-well plates containing 1 mL of prewarmed MTT solution (0.5 mg/mL in growth medium), and incubated for 2 h at 37°C. Then, the cells were washed two times with PBS. The blue formazan precipitate was extracted from the mitochondria using 0.5 mL of dimethyl sulfoxide on a shaker at room temperature for 30 min. Next, 200 *μ*L of this solution was transferred to a 96-well plate, and the absorption at 540 nm (OD_540_) was determined spectrophotometrically. The median OD_540_ of control tissue (cell cultures exposed to the polyvinylsiloxane impression material) was set to represent 100% viability. Results of the cytotoxicity experiments were expressed as a percentage of matching control tissue, and differences between median values were statistically analyzed using the Kruskal-Wallis one-way analysis of variance (ANOVA) and the Mann-Whitney *U* test (*α* = 0.05; SPSS version 13.0; SPSS, Chicago, IL).

## 3. Results

**Figure 1:**
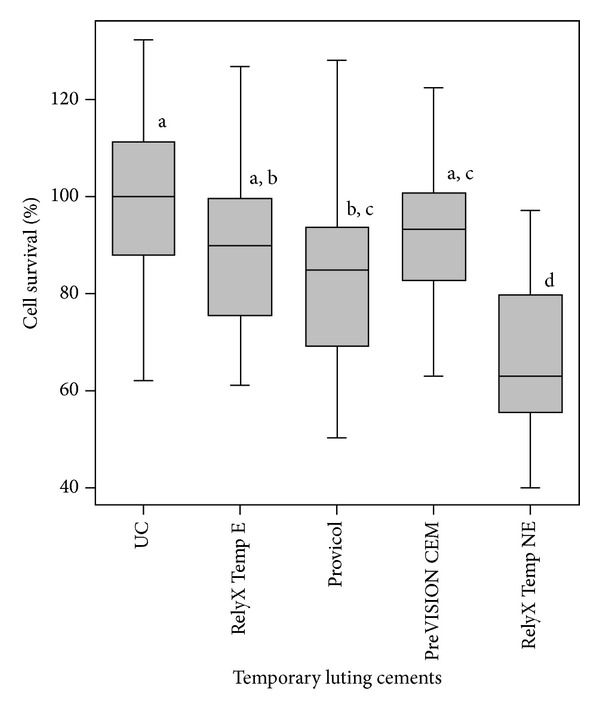
Cytotoxicity of temporary luting cements on two-dimensional cultures of transfected bovine pulp-derived cells. The cell cultures were exposed for 24 h, and cellular survival in treated and untreated cell cultures was determined (*n* = 24). The different small letters over each box indicate significant differences in cytotoxicity. UC, untreated controls.

The results of the cytotoxicity studies with two-dimensional cultures of bovine dental pulp-derived cells are summarized in Figure 1. The average percentage of cell viability after exposure to the extracts of RelyX Temp E, Provicol, PreVISION CEM, and RelyX Temp NE was 89.1%, 84.9%, 92.3%, and 66.8%, respectively. RelyX Temp NE significantly decreased the cells' viability compared to the control group (*P* = 0.000), and the difference in survival rates between RelyX Temp NE and all other tested temporary luting cements was statistically significant (*P* < 0.01). Provicol was the other temporary luting cement showing some cytotoxic effects on bovine dental pulp-derived cells. Provicol decreased the cells' viability compared to the control group (*P* = 0.04); however, the difference in survival rates among Provicol, RelyX Temp E, and PreVISION CEM was not statistically significant (*P* > 0.05). RelyX Temp E and PreVISION CEM were not cytotoxic on bovine dental pulp-derived cells (*P* > 0.05).

**Figure 2:**
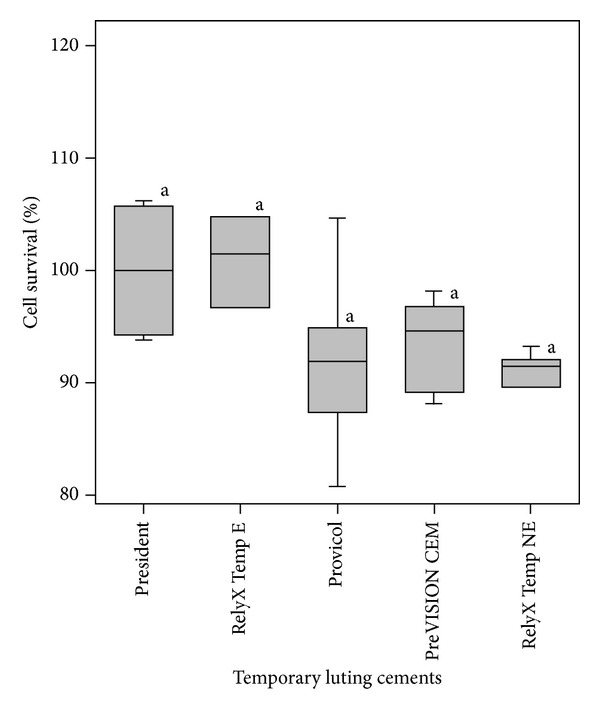
Cytotoxicity of temporary luting cements on three-dimensional cultures of transfected bovine pulp-derived cells (dentin barrier test). Data are expressed as percentage of the negative control (President) cultures (*n* = 10). The same small letters over each box indicate no significant differences in cytotoxicity.

The cytotoxicity of the temporary luting cements was also determined in three-dimensional cell cultures introduced in a dentin barrier test device (Figure 2). None of the temporary luting cements significantly reduced cell survival when compared with the negative control in dentin barrier test (*P* > 0.05). Exposure of the cell cultures to Provicol, PreVISION CEM, and RelyX Temp NE led to 91.9%, 93.5%, and 90.6% cell survival. On the contrary, slightly increased cell vitality was observed with RelyX Temp E (101.5%).

## 4. Discussion

In the present study, the cytotoxic reaction of transfected bovine pulp-derived cells after exposure to temporary luting cements was evaluated by two *in vitro* test methods. The hypothesis of this study can be partly accepted because two of the tested temporary luting cements (Rely X Temp NE and Provicol) were cytototoxic to the two-dimensional monolayer cultures of bovine pulp-derived cells. On the other hand, none of the temporary luting cements were cytotoxic to the three-dimensional cultures of bovine pulp-derived cells (dentin barrier test). 

According to national and international regulations, dental materials have to be evaluated for biocompatibility before being applied to patients. For this purpose, animal experiments or cell culture tests are available. Animal experiments to test the cytotoxicity of dental materials are time consuming, expensive, and subject to extensive public discussions. *In vitro* cytotoxicity test has the advantage of easy control of experimental factors that are often problematic when performing experiments *in vivo*. *In vitro* methods are reproducible, cost effective, relevant, and suitable for the evaluation of basic biological properties of dental materials [10, 17]. Different *in vitro* test methods and cell lines have been used to determine dental materials' cytotoxicity. Primary pulp cells are closely related to their original tissue and have a nearly unchanged metabolic state relative to their original tissue. Thus, an *in vivo* situation may be better simulated by primary pulp cell cultures [18]. However, the isolation of primary cells from target tissues is labor intensive and time consuming, and the resulting cell numbers are often very low compared to the almost unlimited number of cells obtained from continuous cell lines. Additionally, primary pulp cells in cultures have a limited capacity to divide and soon reach a nonproliferative state, probably due to chromosomal alterations. This cellular senescence dramatically limits the availability of cells derived from pulp to investigate responses to material exposure. To overcome these shortcomings, Thonemann and Schmalz introduced an immortalized bovine dental pulp-derived cell line. This cell line, which is also used in the present study as the target cell line, was shown to have similar biochemical characteristics as primary dental pulp cells [15, 16, 19]. 

Adequate contact between cells and test material is crucial for cell cytotoxicity testing. To simulate the *in vivo* situation for temporary luting cements, *in vitro* pulp chambers have been used in the present study, introducing dentin as a barrier between test materials and target cells. Transformed bovine dental pulp-derived cells were grown as three-dimensional cultures and included in a dentin barrier test device to mimic the interactions between the target cells and test materials that occur *in vivo* [9, 16, 20–22]. An additional advantage of this artificial pulp chamber was thought to be the possibility of perfusing the pulpal part with a nutrition medium, thus simulating *in vivo* pulpal blood flow [9].

In the present study, dentin proved to be protective for temporary luting cements. Temporary luting cements were not cytotoxic on bovine pulp-derived cells, although only 0.5 mm thick dentin was used as a barrier. In accordance with our data, dentin has been shown to reduce the toxicity of certain dental materials [23]. Permeability measurements have shown dentin to be a partial perfusion/diffusion barrier [24]. Dentin acts as a diffusion and adsorption barrier, thus reducing the concentration of eluted substances that reach the pulp and possibly cause tissue reactions [25, 26]; it is also considered a solid buffer, neutralizing protons derived from acids [23]. Dentin has also been shown to act as a barrier, decreasing the elicited cytotoxicity with increasing thickness [27]. 

Zinc oxide eugenol is one of the major components of a number of temporary cements because of its bacteriostatic effect, low cost, ease of removal, and good sealing ability [28]. This material elicits a strong reaction *in vitro* [17], but it does not damage the pulp in the same way when applied to the dental cavity with an intact dentin layer *in vivo* [16]. It has been postulated that the dentin layer is responsible for the different results obtained *in vivo* and *in vitro*. Dentin has the ability to absorb eugenol and zinc [27]. The results of this study suggest that eugenol-containing temporary cement (RelyX Temp E) is not cytotoxic to pulp-derived cells, even if a dentin barrier does not exist.

However, the existence of a dentin barrier over the pulp cells seems to be more critical for eugenol-free temporary cements. Although Rely X Temp NE and Provicol were found to be nontoxic in the dentin barrier test, they significantly decreased the survival rate of pulp-derived cells in two-dimensional cultures. Provicol is a calcium-hydroxide-based temporary luting cement. Recently, a number of studies have focused on the biocompatibility of calcium-hydroxide-based endodontic sealers and pulp-capping materials. Van Landuyt et al. reported that the calcium-hydroxide-based root canal sealer Calcicur was not genotoxic to human gingival fibroblasts [28]. Hirschman et al. evaluated three of the current direct pulp-capping agents and reported that among them only Dycal was shown to have a statistically significant cytotoxic effect on adult human dermal fibroblasts [29]. Camargo et al. analyzed cellular reactions to the pulp capping materials. It appeared that only the calcium hydroxide preparation Hydro C was cytotoxic on human pulp derived cells [30]. 

RelyX Temp NE and Provicol both include zinc oxide and rosin. Combinations of rosin and zinc are used in dentistry as components of periodontal dressings and cements and as root canal sealers. Sunzel et al. [31] showed that rosins and pure resin acids had a strong dose-related cytotoxicity that was inhibited by increased zinc concentrations. These materials showed cytotoxic effects in this study as well. Another ingredient of RelyX Temp NE is acrylic acid, and Kurata et al. [32] showed that fibroblast growth decreased upon exposure to acrylic acid as acid increased. RelyX Temp NE was found to be the most toxic temporary luting cement on two-dimensional cultures in the present study. On the other hand, PreVISION CEM also contains zinc oxide, and this material was not cytotoxic to bovine pulp-derived cells in both tests.

## 5. Conclusion

The findings presented here imply that biologically active substances may be released from temporary luting cements and eventually damage pulp-derived cells. However, biologically active substances released from temporary luting cements may not influence the dentine-pulp complex if the residual dentine layer is at least 0.5 mm thick. 

## Figures and Tables

**Table 1:** Test materials, lot numbers, and compositions.

Material	Composition
Provicol Eugenol-free temporary luting cement with calcium hydroxide Lot: base: 770518; catalyst: 770520 Voco GmbH, Cuxhaven, Germany	Zinc oxide, magnesium oxide, calcium hydroxide, fatty acids, vegetable oils, polyglycols, rosin, fumed silica

PreVISION CEM One-component, eugenol-free, silicone-resin-based temporary cement Lot: 255475 Heraeus Kulzer GmbH & Co. KG, Hanau, Germany	Zinc oxide, pyrogenic silica, titanium tetrapropanolate, fillers/binders

RelyX Temp NE Temporary cement-zinc oxide noneugenol Lot: base: 317895; catalyst: 318276 3M ESPE AG, Seefeld, Germany	Catalyst: rosin, reaction products with acrylic acid, nonanoic acid, silane treated silica Base: zinc oxide, white mineral oil (petroleum), petrolatum

RelyX Temp E Temporary cement-zinc oxide-eugenol Lot: base: 239526; catalyst: 239869 3M ESPE AG, Seefeld, Germany	Modified rosin, hydrogenated rosin eugenol, silane treated silica, oleic acid, 2,6-di-tert-butyl-p-cresol
